# Another Country Doctor Heard from

**Published:** 1917-05

**Authors:** Emmett E. Irwin

**Affiliations:** Boles, Ark.


### Another Country Doctor Heard From.

*Editor Texas Medical Journal, Austin, Texas.*

DEAR EDITOR: I have read the article in your February number by Dr. C. T. Bradford, Klondike, Texas, under the caption, "The Experience of a Country Practice Necessary for a Complete Medical Training," three times, and each time with increasing interest.

It seemed as though he had watched the good Samaritan endorse my note at the bank, and then when I graduated and began to practice against able and experienced competition, he had seen the runner on horseback call me out in the dead hours of that same night mentioned, and heard me ask the runner the questions referred to, in an effort to make a diagnosis and outline the treatment before I reached the house.

In my fancy I can now see him watching me through that crack by the chimney as I entered the door and stood there a moment watching eclampsia shake my patient into unconsciousness. The oil lamp was on the job and the old woman holding it would cast a shadow exactly on the spot I wanted to illumine.

I am sure he heard me take that awful "cussing" from the women of the house when I had to quarantine the salaried man, who was the father of six children, but he forgot to mention about the time little Johnnie cut his foot with the ax and his grandmother stopped the hemorrhage with "cobwebs and smut" (soot) before we got there. After washing it about half an hour the wound was still unclean, but healed on first intention. Or how our inventive intuition rambled, when the four-year-old boy swallowed a small nail, and fetched the idea of giving him half-baked biscuit dough to envelop the nail and castor oil to eliminate the "wad."

The country doctor at times is forced to diagnose without diagnostic facilities, treat with what he has on hand and not what he knows about, do surgical work "knee deep" in filth, and yet he is successful.

But we are highly appreciative of our city brother, and thankful for his advices through the press, and know that he must sometimes wish to get out in the country and help me mix sport with our work, while we sometimes long for time enough to visit in the city.

EMMETT E. IRWIN,
Boles, Ark.

